# Sex as a determinant of disease severity and clinical outcome in febrile children under five presenting to a regional referral hospital in Uganda

**DOI:** 10.1371/journal.pone.0276234

**Published:** 2022-10-21

**Authors:** Chloe R. McDonald, Andrea M. Weckman, Emma Richardson, Michael T. Hawkes, Aleksandra Leligdowicz, Sophie Namasopo, Robert O. Opoka, Andrea L. Conroy, Kevin C. Kain

**Affiliations:** 1 SAR Laboratories, Sandra Rotman Centre for Global Health, University Health Network-Toronto General Hospital, Toronto, Canada; 2 Department of Laboratory Medicine and Pathobiology, Faculty of Medicine, University of Toronto, Toronto, Canada; 3 Clinical Epidemiology & Biostatistics Department, Faculty of Health Sciences, McMaster University, Hamilton, Canada; 4 Division of Pediatric Infectious Diseases, University of Alberta, Edmonton, Canada; 5 Interdepartmental Division of Critical Care Medicine, Department of Medicine, University of Toronto, Toronto, Canada; 6 Department of Paediatrics, Kabale Regional Referral Hospital, Kabale, Uganda; 7 Department of Paediatrics and Child Health, Mulago Hospital and Makerere University, Kawempe, Kampala, Uganda; 8 Department of Pediatrics, Indiana University, School of Medicine, Indianapolis, Indiana, United States of America; 9 Toronto General Hospital Research Institute, University Health Network, Toronto, Canada; 10 Tropical Disease Unit, Division of Infectious Diseases, Department of Medicine, University of Toronto, Toronto, Canada; Shandong Public Health Clinical Center: Shandong Provincial Chest Hospital, CHINA

## Abstract

Sex and gender are well-established determinants of health in adult and adolescent populations in low resource settings. There are limited data on sex as a determinant of host response to disease and clinical outcome in febrile children in sub-Saharan Africa, where the risk of infection-related mortality is greatest. We examined sex differences and gender biases in health-seeking behavior, clinical care, biological response to infection, or outcome in a prospective observational cohort of febrile children under 5 years of age presenting to a regional referral hospital in Jinja, Uganda. Main outcomes (stratified by sex) were disease severity at presentation measured by clinical and biological parameters, clinical management (e.g., time to see a physician, treatment by diagnosis), and disease outcome (e.g., mortality). Clinical measures of disease severity included Lambaréné Organ Dysfunction Score (LODS), Signs of Inflammation in Children that Kill (SICK), and the Pediatric Early Death Index for Africa (PEDIA). Biological measures of disease severity were assessed using circulating markers of immune and endothelial activation associated with severe and fatal infections. Differences in outcome by sex were analyzed using bivariate analyses with Bonferroni correction for multiple comparisons. In this cohort of febrile patients admitted to hospital (n = 2049), malaria infection was common (59.2%). 15.9% of children presented with severe disease (LODS score ≥ 2). 97 children (4.7%) died, and most deaths (n = 83) occurred within 48 hours of hospital admission. Clinical measures of disease severity at presentation, clinical management, and outcome (e.g., mortality) did not differ by sex in children under five years of age. Host response to infection, as determined by endothelial and inflammatory mediators (e.g., sTREM1, Ang-2) quantified at hospital presentation, did not differ by sex. In this cohort of children under the age of five, sex was not a principal determinant of disease severity at hospital presentation, clinical management, disease outcome, or biological response to infection (p-values not significant for all comparisons, after Bonferroni correction). The results suggest that health seeking behavior by caregivers and clinical care in the hospital setting did not reflect a gender bias in this cohort.

## Introduction

Globally, an estimated 5.9 million children per year die before they reach the age of five [1]. Despite ongoing efforts to reduce global mortality, most of these children die in resource-constrained regions of Sub-Saharan Africa and South Asia. Numerous social determinants including geography, household income, social status, and parents’ level of education influence health outcomes for both boys and girls [2]. Girls often face systemic gender bias on top of socio-economic barriers to health access [2–4]. Most research on biological sex differences and gender bias has been conducted about adults and adolescents and has not extensively documented sex differences or gender-based health inequities in pediatric populations less than 5 years of age. Existing population-level evidence indicates that males carry a higher global burden of under-five mortality [5, 6], but that this effect is strongly dependent on specific region [7]. Increased mortality for male children is thought to be due to a biological, X-chromosome related survival advantage conferred to female children that is eliminated in regions where females experience high rates of gender bias [6]. For example, research conducted about South Asian children under five suggests that less money is spent on the medical care of girls, that female children are less likely be immunized, or to receive access to medical care, that girls are brought to health care facilities at later stages of illness, have lower rates of hospitalization for severe disease, and ultimately have higher rates of mortality [2, 8, 9]. It is estimated that childhood under-five mortality in India could be reduced by 20% if girls had the same mortality rates as boys [2]. To-date few studies have examined how sex and gender influence health seeking behavior of caregivers and health outcomes in children under five in sub-Saharan Africa, where mortality is highest [1].

Fever is one of the most common symptoms in children under 5 years presenting to healthcare facilities in low and middle-income countries, with a mean of 5.9 fever episodes per child per year [10]. Current evidence suggests that sex can modify the host immune response to infectious diseases. Sex-mediated differences in the host immune response begin at conception and are mediated by both the X and Y-chromosomes [11–13]. The X chromosome expresses genes that are critical immune system regulators, including Toll-like receptors, cytokines, and genes regulating T and B-cell activity, while the Y chromosome encodes several immune response genes that are only expressed in males [12, 13]. Random silencing of most alleles on one X chromosome during embryogenesis and the resulting polymorphisms in X-linked genes may result in cellular advantages for females in the host immune response [11, 14, 15]. Sex hormones also impact the innate immune system and modulate immuno-competence [11]. For example, estradiol influences the cellular immune response and can reduce the release of anti-inflammatory cytokines and increase the anti-inflammatory and chemotactic activity of neutrophils [16–18]. Most of the research on sex-differences in host immune response has examined disease severity and outcome in adults, but the genetic and hormonal origins of sex-mediated differences suggest they also impact the host immune response in children [19].

Sex-associated differences in the pathophysiology of severe infections are not well studied in children, particularly in regions of Sub-Saharan Africa, where gender-based health inequities in care-seeking and health access may intersect with sex differences in biological pathways that underlie disease severity. Here we examined a prospective cohort of children under five presenting with fever to a regional referral hospital in Jinja, Uganda. We examined gender as a social determinant of health-seeking behavior by the child’s caregiver, as well as the impact of sex on biological pathways (i.e. endothelial and immune activation) that directly contribute to the pathobiology of severe malaria and other life-threatening infections [20–25].

## Materials and methods

### Study setting and cohort

This is a secondary analysis of a prospective observational fever cohort study conducted in Jinja, Uganda, which examined markers of inflammation and endothelial injury in febrile children aged two months to five years admitted to Jinja Regional Referral Hospital between February 2012 and August 2013, as described [26, 27]. Jinja Regional Referral Hospital is a public healthcare facility in mid-eastern Uganda that, at the time of this study, served a catchment area of three million people across 12 districts. The pediatric unit had 100 beds, five pediatricians, and averaged 650 admissions/month.

The primary outcome of the study was all-cause in-hospital mortality. Inclusion criteria were: age two months to five years; parent’s report of fever within the last 48 hours, or axillary temperature greater than 37.5°C; hospital admission according to the admitting physician’s judgment. Children with gastroenteritis/diarrhea alone and no other signs of infection were excluded. Patients were managed in accordance with national standard of care guidelines for the treatment of malaria, pneumonia, sepsis, meningitis, respiratory distress, anemia, and hypoglycemia. Outcome was assessed by daily review of clinical and laboratory features. Clinical data was collected at enrollment, during hospitalization and at discharge. Laboratory tests at admission included oxygen saturation, blood microscopy for malaria diagnosis, three-band rapid diagnostic test for malaria diagnosis, glucose, lactate, hemoglobin, and HIV antibody. Disease severity scores, including the Blantyre Coma Score (BCS), Signs of Inflammation in Children that Kill (SICK), Lambaréné Organ Dysfunction Score (LODS), Pediatric Early Death Index for Africa (PEDIA), were computed based on physical exam findings from all children at admission [26]. The current study excluded any participants with unknown sex or age (n = 35) (S1 Fig in S1 File).

### Definitions

Based on the World Health Organization definition of infant mortality (mortality in children ≤1 year) [28], the data are presented for children ≤1 year (2 to ≤12 months of age) and for children >1 year (>12 to 60 months of age). We hypothesized that potential gender-bias by caregivers towards female children would manifest as girls being brought to hospital at later stages of illness, with evidence of more severe disease. We also hypothesized that gender-based inequities in hospital care would result in differences in clinical outcomes between boys and girls. Finally, we hypothesized that sex-based differences in host response to infection (e.g., immune and endothelial activation) would be associated with disease severity and outcome. To test these hypotheses, we used a clinically validated score of infection-related severity (LODS score). A cut off of LODS <2 compared with LODS ≥2 was used based on evidence that a significant increase in mortality is observed in children with LODS ≥2 [26]. Here, gender bias refers to differences in health seeking behavior for children by primary caregivers and/or differences in clinical management based on social constructions of gender (e.g. potential differences in gender value and/or norms) [29]. It was inferred from differences between boys and girls in disease severity at time of presentation, clinical management (e.g., access to treatment), and outcome. Sex differences refer to biological differences in host immune response (markers of immune and endothelial activation) by sex.

### Quantifying markers of immune and endothelial activation

For analyte quantification, a plasma sample was collected at clinical presentation. Markers of inflammation and endothelial dysfunction linked to infection-related disease severity and mortality were quantified by a multiplex platform (Luminex®, R&D Systems) [27]. Markers of inflammation included interleukin 6 (IL-6), interleukin-8/C-X-C motif chemokine ligand 8 (IL-8/CXCL8), interferon-gamma-inducible protein-10/C-X-C motif chemokine ligand 10 (IP-10/CXCL10), chitinase-3-like-1 protein (CHI3L1), soluble triggering receptor expressed on myeloid cells-1 (sTREM-1), soluble tumor necrosis factor receptor-1 (sTNFR-1), and Granzyme B. Markers of endothelial activation included vascular cell adhesion molecule (sVCAM-1), soluble intracellular adhesions molecule-1 (sICAM-1), angiopoietin-1 (Angpt-1), angiopoietin-2 (Angpt-2), and soluble fms-like tyrosine kinase-1 (sFlt-1). Eight samples on each plate were performed in duplicate to ensure intra-assay consistency [27]. PCT (RayBiotech) and CRP (R&D Systems) were quantified using single analyte ELISAs. Biomarkers concentrations were log_e_-transformed and values below the limit of detection were assigned a value of 1/3 of the lowest point on the standard curve. All protein quantification was performed blinded to clinical outcome or sex.

### Statistical analysis

Statistical analysis was performed using STATA v12 (StataCorp, TX) and R version 3.2.1 (R Foundation for Statistical Computing) software. Descriptive data were summarized using median [interquartile range, IQR] or n (%). The proportion of male versus female participants was compared using a binomial probability test. Baseline characteristics were compared between male and female participants using the Pearson Chi-square test or Fisher’s exact test where appropriate. Concentrations of biomarkers, laboratory and clinical parameters showed deviation from normality (Shapiro-Wilks p < 0.05), and therefore were non-parametrically tested for differences between male and female participants using the Wilcoxon rank-sum test. Since previous studies have reported associations between mediators of immune/endothelial activation and disease severity and mortality [30–33], we also examined concentrations of these analytes stratified by sex and disease severity (as indicated by LODS <2 and LODS ≥2) or in-hospital mortality. All P values were two-sided and values of <0.05 were considered statistically significant. Adjustment of the P value for multiple comparisons was performed using the Bonferroni correction. No statistically significant differences were reported by bivariate analysis; therefore, we did not perform subsequent multivariate analysis.

A sample size calculation indicated that we would need at least 1,628 patients to detect a clinically significant difference of 10% in any proportion between males and females, with 80% power at the alpha = 0.05/38 level of significance. This sample size accounts for Bonferroni correction for family-wise type 1 error rate for comparisons of up to 38 binary variables.

### Ethics

The study had ethical approval from the Ugandan National Council for Science and Technology, Makerere University Research Ethics Committee in Uganda (Kampala, Uganda, REC Protocol # REF 2011–255), and the University Health Network Research Ethics Board (Toronto, Canada, REB: 12-0039-AE). The accompanying parent or caregiver provided written informed consent for all study participants including consent for blood sampling and data collection.

### Inclusivity in global research

Additional information regarding the ethical, cultural, and scientific considerations of this study specific to inclusivity in global research is included in the S1 Checklist.

## Results

### Characteristics of the total study cohort

Of the 2,502 children enrolled in the parent trial, 82% (n = 2,049) met the inclusion criteria for this study. 418 children were transferred, lost to follow-up, or did not have a biomarker sample (unknown outcome), and 35 children did not have a known age and/or sex. A detailed study flow diagram is presented in S1 Fig in S1 File. The median age of children in the total study cohort was 17 months (1.4 years). At enrolment in the trial, 43 children (2.1%) were living with HIV, 59.2% of children were diagnosed with malaria, 16.1% with IMCI pneumonia, and 1.3% with meningitis. 97 children (4.7%) died during hospital admission and most fatalities (n = 83/97, 85.6% of fatalities) occurred within 48 hours of admission (Tables 1–3). In children ≤ 1 year (n = 758) there was no significant difference between the number of female (n = 356, 46.9%) and male (n = 402, 53.0%) participants. In children >1 year of age there was a lower proportion of girls (n = 568, 44.0%) versus boys (n = 723, 56.0%, P = 0.001) (S1 Fig in S1 File).

**Table 1:** Baseline characteristics of study population by sex.

		Sex Stratified					
		≤1 Year of Age (n = 758)			>1 Year of age (n = 1,291)		
	Entire Cohort (n = 2,049)	Female (n = 356)	Male (n = 402)	P Value^2^	Female (n = 568)	Male (n = 723)	P Value^2^
**Demographic** ^**1**^							
Age (months)	17 [9,26]	8[6,10]	8[6,10]	0.858	24[18,36]	24[18,36]	0.334
Length (cm)	73 [65,81]	63 [59,68]	65 [60,70]	0.009	78 [72,86]	79 [73,87]	0.033
Weight (kg)	9 [8,11]	7.35 [6.0, 8]	8 [7,9]	**0.0009**	10 [9,12]	10 [9,13]	**0.0007**
MUAC (cm)	14 [13,15]	13.5 [12.7, 14.5]	13.5 [12.6, 14.5]	0.451	14 [13.5, 15]	14.5 [13.5, 15.5]	**0.0001**
**Clinical History** ^**1**^							
Previous hospitalization	1094 (53.4)	109 (30.6)	161 (40.0)	0.014	379 (66.7)	445 (61.5)	0.089
Chronic illness	134 (6.5)	12 (3.4)	14 (3.5)	0.971	47 (8.2)	61 (8.4)	0.871
Pretreatment							
Antibiotics	796 (38.8)	153 (43.0)	165 (41.0)	0.477	206 (36.3)	272 (37.6)	0.917
Antimalarials	1117 (54.5)	153 (43.0)	208 (51.7)	0.023	331 (58.3)	425 (58.8)	0.677
HIV	43 (2.1)	5 (1.4)	8 (2.0)	0.535	15 (2.6)	15 (2.1)	0.503
**Examination** ^**1**^							
Temperature	37.9 [37, 38.8]	37.9 [37,38.8]	37.9 [37.1, 38.8]	0.992	38 [37, 38.9]	37.8 [36.9, 38.8]	0.048
Heart rate	162 [146, 177]	170 [154, 187]	169 [154, 183]	0.509	160 [146, 174]	155 [138, 170]	**<0.0001**
Respiratory rate	44 [36, 56]	48 [40, 62]	50 [40, 61]	0.961	40 [33, 52]	40 [32, 50]	0.435
Blood Pressure							
Systolic	100 [90, 110]	100 [90, 110]	100 [90, 110]	0.074	100 [90, 100]	100 [90, 100]	0.522
Diastolic	58 [50,60]	55 [50,60]	54 [50,60]	0.717	60 [50, 65]	60 [50,65]	0.354
SpO_2_	98 [96, 99]	98 [96, 100]	98 [96, 99]	0.833	98 [96, 99]	98 [96, 99]	0.417
Methemoglobin (%)	1.5 [0.7, 2.6]	1.5 [0.4, 2.4]	1.4 [0.4, 2.9]	0.446	1.6 [0.9, 2.8]	1.5 [0.8, 2.4]	0.280
Perfusion Index	1.2 [0.78, 1.9]	1.0 [0.68, 1.6]	1.1 [0.75, 1.7]	0.100	1.2 [0.78, 2]	1.2 [0.85, 2]	0.271
Glucose	6.9 [5.8, 8.3]	6.9 [5.9, 8.3]	7.2 [6.2, 8.2]	0.182	7.0 [5.7, 8.7]	6.8 [5.6, 8.2]	0.014
Lactate	2.7 [1.9, 5]	2.8 [1.9, 6.1]	2.7 [1.9, 4.8]	0.366	2.8 [2, 4.8]	2.6 [1.9, 4.9]	0.154
Hemoglobin (g/dL)^3^	4.7 [3.2, 7]	4.7 [3.2, 6.6]	4.8 [3.4, 6.5]	0.764	4.4 [3.1, 6.8]	4.8 [3.4, 7]	0.160

^1^Data are presented as median [interquartile range] or frequency (percent) as appropriate

^2^Results of Wilcoxon rank-sum test or chi-squared test as appropriate; bolded values indicate P < 0.001 after Bonferroni correction (P = 0.05/38 tests)

^3^Data available for 808 children. Abbreviations: middle-upper arm circumference (MUAC); oxygen saturation (SpO_2_).

**Table 2:** Disease severity at presentation and diagnosis by sex.

		Sex Stratified					
		≤1 Year of Age (n = 758)			>1 Year of age (n = 1,291)		
	Entire Cohort (n = 2049)	Female (n = 356)	Male (n = 402)	P Value^2^	Female (n = 568)	Male (n = 723)	P Value^2^
BCS	5 [5,5]	5 [5,5]	5 [5,5]	0.254	5 [5,5]	5 [5,5]	0.756
LODS	0 [0.1]	0 [0.1]	0 [0.1]	0.439	0 [0.1]	0 [0.1]	0.452
SICK	1.7 [0.9, 2.4]	2.2 [1.2, 2.8]	2.2 [1.2, 2.7]	0.153	1.7 [0.5, 2.1]	1.5 [0.5, 2.1]	0.031
PEDIA	0 [0,2]	0 [0,3]	0 [0,2]	0.987	0 [0,2]	0 [0,2]	0.555
**Diagnosis** ^**1**^							
Malaria	1213 (59.2)	211 (59.3)	226 (56.2)	0.433	340 (59.9)	436 (60.3)	0.797
Pneumonia	329 (16.1)	72 (20.2)	89 (22.1)	0.589	87 (15.3)	81 (11.2)	0.031
Meningitis	27 (1.3)	5 (1.4)	2 (0.5)	0.188	10 (1.8)	10 (1.4)	0.592

^1^Data are presented as median [interquartile range] or frequency (percent) as appropriate

^2^Results of Wilcoxon rank-sum test or chi-squared test as appropriate; P < 0.003 would be considered significant after Bonferroni correction (P = 0.05/16). Abbreviations: Blantyre Coma Score (BCS); Lambaréné Organ Dysfunction Score (LODS); Pediatric Early Death Index for Africa (PEDIA); Signs of Inflammation in Children that Kill (SICK).

**Table 3:** Quality of clinical care, treatment, and clinical outcome by sex.

		Sex Stratified					
		≤1 Year of Age (n = 758)			>1 Year of age (n = 1,291)		
	Entire Cohort (n = 2049)	Female (n = 356)	Male (n = 402)	P Value^2^	Female (n = 568)	Male (n = 723)	P Value^2^
**Quality of Care**							
Time to see physician (hours)	2.58 [1,4.17]	2.32 [1,4]	2.50 [1.25,4.42]	0.050	2.60 [1,4]	3.67 [1,4.25]	0.580
Missing essential supplies	141 (6.9)	17 (48)	24 (6.0)	0.483	42 (7.4)	58 (8.0)	0.664
Length of hospital stay (days)	2 [2,4]	3 [2,4]	3 [2,4]	0.376	2 [2,4]	2 [2,4]	0.216
**Treatments**							
Glucose	476 (23.2)	84 (23.6)	89 (22.1)	0.587	121 (21.3)	171 (23.7)	0.309
Intravenous fluids	250 (12.2)	55 (15.5)	61 (15.2)	0.849	56 (9.9)	78 (10.8)	0.583
Transfusion	686 (33.5)	128 (36.0)	139 (34.6)	0.505	191 (33.6)	228 (31.5)	0.476
Antibiotic							
Ceftriaxone	1063 (51.9)	186 (52.3)	216 (53.7)	0.839	301 (53.0)	360 (49.8)	0.283
Other	923 (45.1)	187 (52.5)	205 (51.0)	0.615	238 (41.9)	293 (40.5)	0.620
Antimalarial							
Quinine	1419 (69.3)	239 (67.1)	280 (69.7)	0.462	400 (70.4)	500 (69.2)	0.677
Other	383 (18.7)	52 (14.6)	55 (13.7)	0.658	115 (20.3)	161 (22.3)	0.387
Oxygen	156 (7.6)	42 (11.8)	36 (9.0)	0.170	33 (5.8)	45 (6.2)	0.758
**Outcome**							
Absconded	346 (16.9)	69 (19.4)	79 (19.7)	0.269	85 (15.0)	113 (15.6)	0.792
Transfer	34 (1.7)	6 (1.7)	8 (2.0)	0.691	10 (1.8)	10 (1.4)	0.585
Death	97 (4.7)	22 (6.2)	19 (4.7)	0.377	20 (3.5)	36 (5.0)	0.202
Death in first 48 hours	83 (4.1)	18 (5.1)	18 (4.5)	0.709	17 (3.0)	30 (4.2)	0.271

^1^Data are presented as median [interquartile range] or frequency (percent) as appropriate

^2^Results of Wilcoxon rank-sum test or chi-squared test as appropriate; P < 0.002 would be considered significant after Bonferroni correction (P = 0.05/28).

### Clinical parameters at presentation based on sex

Demographic and clinical data at presentation, disaggregated by sex, are presented in Table 1. As expected, sex-based differences were observed in length (median: 63 cm [girls] vs. 65 cm [boys], P = 0.009) and weight (median: 7.35 kg [girls] vs. 8 kg [boys], P = <0.001) in children ≤1 year, and in length (median: 78 cm [girls] vs. 79 cm [boys], P = 0.033), weight (median (IQR): 10 kg (9, 12) [girls] vs. 10 kg (9,13) [boys], P < 0.001), middle-upper arm circumference (MUAC) (median: 14 [girls] vs. 14.5 [boys], P < 0.001) and heart rate (median: 160 bpm [girls] vs. 155 bpm [boys], P < 0.001) in children between 1 and 5 years (Table 1). Differences in weight, MUAC, and heart rate remained significant even after adjusting for multiple comparisons. Girls ≤1 year of age were less likely to have been previously hospitalized (30.6% [girls] vs. 40.0% [boys], P = 0.014) or to receive pre-treatment with an antimalarial (43.0% vs. 51.7%, P = 0.023); however, these differences were not significant after controlling for multiple comparisons.

**Fig 1:**
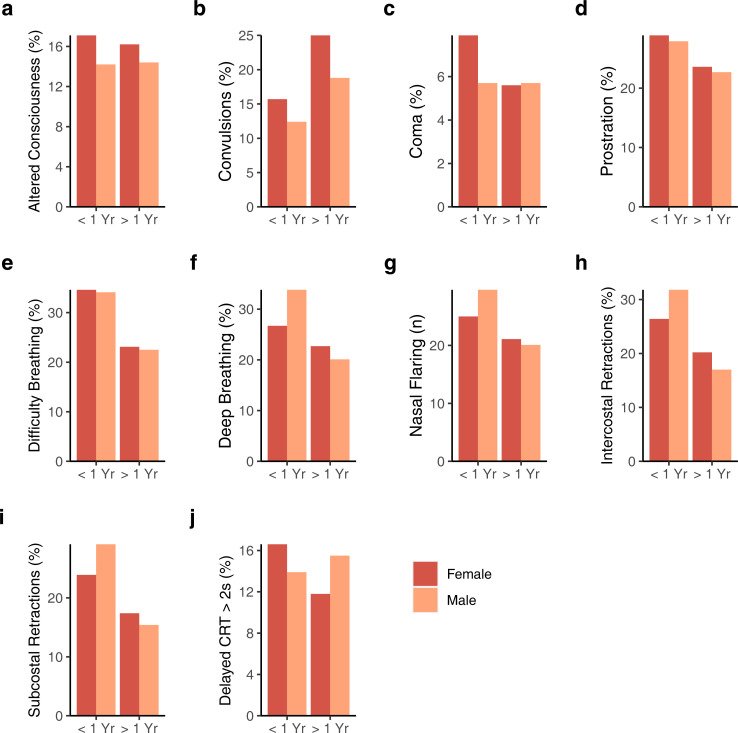
Percentage of participants displaying clinical parameters at presentation by sex in children ≤1 year of age and in children between 1 year and 5 years of age (age range) including: (a) altered consciousness, (b) convulsions, (c) coma, (d) prostration (defined as inability to breastfeed if <6 months, inability to sit up if >6 months), (e) difficulty breathing, (f) deep breathing, (g) nasal flaring, (h) intercostal retractions, (i) subcostal retractions, and (j) delayed capillary refill time (CRT) >2 seconds (2s). No comparisons were significant after controlling for multiple comparisons (P < 0.002 would be considered significant after Bonferroni correction, 0.05/26).

No differences by sex were observed in clinical history including chronic illness, pretreatment with antibiotics, or in number of children with HIV. We observed no differences by sex, in children ≤1 year or >1 year, in temperature, respiratory rate, blood pressure, or clinical variables, including presenting oxygen saturation, glucose, lactate, and hemoglobin after Bonferroni correction (Table 1). Parameters associated with disease severity at presentation, such as labored breathing, convulsions, and coma, did not significantly differ by sex in children ≤1 year of age or in children >1 year of age after adjusting for multiple comparisons (Fig 1, S1 Table in S1 File).

### Disease severity at presentation and diagnosis

To assess for potential gender bias in healthcare seeking behavior by primary care providers, we looked at disease severity at presentation between girls and boys. Girls >1 year of age had a higher SICK score (1.7 (0.5, 2.1) [girls] vs. 1.5 (0.5, 2.1) [boys], P = 0.031) indicative of more severe disease, and a higher prevalence of pneumonia at hospital presentation (15.3% vs. 11.2%, P = 0.031) compared with boys >1 year, but these differences were not significant after Bonferroni correction. In children ≤1 year of age or in children >1 year of age we did not observe differences in disease severity scores, including BCS, LODS, SICK, and PEDIA or in the frequency of diagnosis of malaria, pneumonia, or meningitis (Table 2).

### Treatment and clinical outcome

To examine potential gender biases introduced by healthcare workers, we examined clinical quality of care, treatment, and outcome between girls and boys after hospital admission. In children ≤1 year of age or in children >1 year of age, no differences by sex were observed in access to treatment, including time to see a physician, quality of care, or type of treatment provided (Table 3). There was no difference in the number of deaths, or in deaths within the first 48 hours, between female and male patients in children ≤1 year of age or in children >1 year of age (Table 3). When we examined treatment by diagnosis (malaria or pneumonia) we observed reduced use of antibiotics (other than ceftriaxone) in girls >1 year of age diagnosed with pneumonia (51.7% of girls received antibiotics vs. 70.4% of boys, P = 0.01) (S2 Table in S1 File). We also observed reduced use of quinine in girls ≤1 year of age (84.1% vs. 92.7% in boys, P = 0.013) and increased use in girls >1 year of age (87.9% vs. 80.8% in boys, P = 0.021) that were diagnosed with malaria (S2 Table in S1 File). None of these observations were significant after controlling for multiple comparisons. When we examined differences in treatment based on disease severity at presentation, a higher percentage of girls ≤1 year of age with more severe disease (LODS ≥2) received treatment with oxygen in comparison to boys with more severe disease (48.5% [girls] vs. 25.6% [boys], P = 0.003). This result was not significant after Bonferroni correction. We did not observe any other differences in treatment provided to female versus male patients with respect to disease severity as indicated by their LODS score (S3 Table in S1 File).

### Concentrations of markers of endothelial and immune activation

To examine sex-based differences in biological host response to infection, we analyzed markers of endothelial and immune activation by sex. Compared with boys ≤1 year of age, girls ≤1 year had elevated plasma concentrations of sFlt-1 (419.9 pg/mL [girls] vs. 383.6 pg/mL [boys], P = 0.048) and CXCL8 (537.1 pg/mL vs. 469.8 pg/mL, P = 0.034) at hospital presentation, and boys >1 year of age had higher plasma concentration of Angpt-1 (2.3 ng/mL [girls] vs. 2.7 ng/mL [boys], P = 0.003) and CHI3L1 (46.1 ng/mL vs. 50.8 ng/mL, P = 0.025) compared with girls >1 year (Table 4). However, after controlling for multiple comparisons, there were no significant differences by sex in plasma concentrations of markers of endothelial or immune activation. We did not observe any significant differences in analyte concentrations by sex based on disease severity at presentation, or between children who survived or died in-hospital, after Bonferroni correction (S4, S5 Tables in S1 File).

**Table 4:** Analyte concentrations in children with known outcome stratified by sex.

		Sex Stratified					
		≤1 Year of Age (n = 758)			>1 Year of age (n = 1,291)		
	Entire Cohort (n = 2049)	Female (n = 356)	Male (n = 402)	P Value^2^	Female (n = 568)	Male (n = 723)	P Value^2^
**Endothelial Activation**							
sICAM-1 (ng/mL)	707.3 [374.2, 1202.5]	678.2 [345.2, 1114.5]	650.4 [362.1, 1112.1]	0.505	719.5 [379.4, 119.9]	746.6 [391.9, 127.5]	0.273
sVCAM-1 (ng/mL)	3951.2 [2332.5, 6718.1]	3839.7 [2216.6, 7024.5]	3696.2 [2163.4, 6045.4]	0.604	4203.8 [2419.6, 7115.5]	4000.0 [2340.3, 6631.6]	0.308
Angpt-2 (ng/mL)	8.2 [5.3, 13.8]	9.2 [6.1, 14.8]	8.8 [5.9, 14.1]	0.460	7.7 [5.1, 13.6]	7.3 [4.7, 13.3]	0.116
Angpt-1 (ng/mL)	2.4[1.0, 5.8]	2.1 [0.9, 5.7]	2.3 [0.9, 6.2]	0.700	2.3 [1.0, 4.9]	2.7 [1.2, 6.2]	0.003
sFlt-1 (pg/mL)	373.0 [224.4, 764.3]	419.9 [252.1, 962.0]	383.6 [235.7, 734.7]	0.048	379.1 [217.3, 794.4]	335.6 [212.8, 690.5]	0.144
**Inflammation**							
sTFNR1 (ng/mL)	12.0 [7.7, 19.6]	13.5 [7.7, 24.2]	12.8 [7.9, 21.8]	0.360	11.2 [7.5, 18.5]	11.7 [7.8, 18.0]	0.563
CHI3L1 (ng/mL)	42.6 [11.0 100.5]	34.5 [11.0, 83.4]	34.1 [11.0, 77.3]	0.928	46.1 [11.0, 103.5]	50.8 [22.4, 140.4]	0.025
sTREM1 (pg/mL)	570.7 [374.6, 879.9]	618.1 [411.7, 1031.6]	605.2 [402.1, 903.2]	0.502	572.6 [373.5, 887.5]	522.8 [346.7, 811.4]	0.050
CXCL10 (pg/mL)	430.5 [172.0, 1127.5]	537.1 [198.5, 1426.4]	469.8 [176.6, 1136.4]	0.254	423.2 [178.2, 1109.8]	394.4 [147.9, 1007.6]	0.169
CXCL8 (pg/mL)	13.8 [2.5, 35.5]	16.2 [8.0, 40.1]	13.7 [2.5, 31.7]	0.034	12.3 [2.5, 38.3]	12.8 [2.5, 35.9]	0.223
IL6 (pg/mL)	31.6 [5.0, 116.8]	32.1 [5,112.1]	25.5 [5, 102.9]	0.095	37.6 [5, 137.3]	31.3 [5, 114.6]	0.450
Granyzme B (pg/mL)	86.0 [20.0, 255.4]	117.5 [20, 361.7]	117.7 [20, 297.6]	0.240	76.8 [20, 223.3]	66.6 [20, 208.1]	0.361

^1^Data are presented as median [interquartile range], ^2^Results of Wilcoxon rank-sum test; P < 0.002 would be considered significant after Bonferroni correction (P = 0.05/24). Abbreviations: angiopoietin-1 (Angpt-1); angiopoietin-2 (Angpt-2); chitinase-3-like-1 protein (CHI3L1); interferon-gamma-inducible protein-10/C-X-C motif chemokine ligand 10 (IP-10/CXCL10); interleukin 6 (IL-6); interleukin-8/C-X-C motif chemokine ligand 8 (IL-8/CXCL8); soluble fms-like tyrosine kinase-1 (sFlt-1); soluble intracellular adhesions molecule-1 (sICAM-1); soluble triggering receptor expressed on myeloid cells-1 (sTREM-1), soluble tumor necrosis factor receptor-1 (sTNFR-1); vascular cell adhesion molecule (sVCAM-1).

## Discussion

In this study we report that clinical parameters indicating disease severity at presentation, in-hospital treatment, diagnosis, and outcome did not differ by sex in a pediatric cohort of febrile children presenting to a regional referral hospital in Jinja, Uganda. Moreover, we did not observe differences in the concentrations of markers of immune and endothelial activation linked to severe and fatal infections, in girls versus boys. Concentrations of these markers did not differ by sex based on disease severity at presentation as determined by a validated clinical scoring system (LODS) [34]. To our knowledge this is the first study to examine sex in relation to biological pathways mediating disease severity in a cohort of children under five. Our results suggest that female and male children with fever syndromes were admitted to the hospital at similar stages of illness, received comparable treatment, and had similar biological responses and clinical outcomes. Collectively we found no evidence of sex differences or gender bias as a determinant of health seeking behaviors, clinical outcome, biological response to infection, or mortality in this cohort of febrile children under the age of five. Collectively, our data highlight a positive outcome in the context of gender equality–once admitted to hospital, children in this setting seem to be receiving care without sex and gender disparities.

We observed a lower proportion of female patients admitted to hospital compared with male patients in children over one year of age. This may indicate a gender bias in that fewer girls are being brought to the hospital in children >1 year of age, or fewer girls are being admitted to hospital when ill. Future studies should include an outpatient comparison group to determine if these differences reflect a disparity in total number of male/female children being brought to hospital, or a sex-difference in hospital admission. Alternatively, greater vaccine efficacy (i.e. stronger humoral response) in girls may mean girls are at a lower risk of infection, as indicated by previous work in this field [35, 36]. As we did not observe differences in biological or clinical parameters of disease severity between girls and boys at presentation, these data suggest that differences in hospital admission may not be a result of gender-based inequalities in health seeking by caregivers at this age, but rather a reflection of the need for care. Additional research is required to confirm or refute these findings and needs to be paired with outpatient and community-level disease severity and mortality rates to determine if there are gender-disparities in care-seeking for febrile children in the community, and consequent disparities in community-level mortality.

There are limited data on sex-associated differences in inflammatory and endothelial activation pathways, especially in children in low and middle-income countries who are at the greatest risk for severe and fatal infections [1]. Endothelial and immune activation contribute to the pathogenesis of life-threatening infections [23, 30]. Mediators of immune and endothelial activation including cytokines and the Angiopoietin-Tie2 signaling axis correlate with severity of illness, multi-organ dysfunction, and mortality in life-threatening infections [20–22, 24, 33]. Our analysis of these activation markers provides biological support for the lack of observed differences in clinical disease severity between girls and boys. If girls were brought to hospital at more advanced stages of illness, we would expect to see increased concentrations of these key mediators of severe infection. We did not observe any differences by sex; supporting our observation that children were brought to the hospital at similar stages of illness. Moreover, we did not find differences in mediator concentrations by sex when we stratified the cohort by LODS score. These data indicate that in young children with severe infections, as indicated by a higher LODS score, there is no differential host response based on sex. Our results support the hypothesis that sex is not a critical factor in infection-induced inflammatory and endothelial activation in children under 5 years of age.

This study had a number of strengths including the prospective design, detailed clinical data on disease severity, and the ability to examine multiple clinical parameters as well as biological responses to assess disease severity. However, this study has limitations. It is a secondary analysis of a cohort of children enrolled in a previously completed study. Therefore, while adequately powered to determine if there were sex differences in biological responses (i.e., disease severity markers), it was not sufficiently powered to analyze sex-stratified mortality as a primary outcome. As this study enrolled only febrile children admitted to hospital, we were unable to determine gender biases in care-seeking for non-febrile reasons, or outpatient healthcare and outcome, and limited in our ability to estimate gender differences in hospital admission rates with respect to total number of children presenting to hospital (including outpatients). Additional studies are required to examine mortality and how healthcare provision and mortality in outpatient and community settings is related to mortality in-hospital with respect to sex differences and gender biases. The study site was a regional referral hospital with a large catchment area. While the resultant cohort would likely be representative of the region, further studies would be needed to validate these findings in other regions of sub-Saharan Africa. Moreover, we found multiple trends toward gender bias (e.g., lower rates of antibiotic and quinine administration in girls) that were no longer significant after correcting for multiple comparisons, indicating that despite a large sample size we may have been limited in our power to investigate so many dependent variables. While it is necessary to address the potential for false positives when performing many simultaneous comparisons, we employed a relatively conservative statistical correction (Bonferroni), which must be considered in the interpretation of our findings. The analysis included participants who enrolled in the study and had a known primary outcome (i.e., all-cause in-hospital mortality). Therefore, it is possible that the cohort was influenced by gender biases in parent or immediate caregiver decisions to enroll their child in this study. In addition, the original prospective observational cohort was not designed to directly assess gender bias as a determinant of health. Additional focused studies are required to further answer the question of how biological sex differences and gender biases impact health outcomes in children under five in sub-Saharan Africa.

### Conclusions

Addressing sex and gender-based inequities in health access and outcomes requires an understanding of when inequities emerge. The results of this study provide promising preliminary evidence that social determinants of health grounded in sex may not influence disease severity at hospital presentation, diagnosis, treatment, or clinical outcome of children under 5 years in Uganda. This conclusion is supported by biological data indicating that the host response to infection, measured by immune and endothelial activation markers, did not differ by sex at presentation. While our results indicate that potential sex- or gender-based inequities did not impact health outcomes in this pediatric cohort, it is well established that sex- and gender-based inequalities create imbalances in health access for adolescent girls and women in low-resource settings [37]. Therefore, it remains possible that inequities may manifest at a later age, when social and cultural gender norms begin to have a stronger influence. Furthermore, it remains possible that community-level data or a larger cohort designed specifically for this analysis would reveal gender biases that were not evident in our facility-based data. More research is needed in pediatric populations in sub-Saharan Africa to establish when health inequities emerge and where resources should be allocated to promote gender equality in access to healthcare and to improve outcomes.

## Supporting information

S1 Checklist Inclusivity in global research. (DOCX)

S1 File (DOCX)
