# Supplementary material for: Sex as a determinant of disease severity and clinical outcome in febrile children under five presenting to a regional referral hospital in Uganda
Source: PLoS One. 2022 Oct 21;17(10):e0276234. doi: 10.1371/journal.pone.0276234 (PMC9586386; doi:10.1371/journal.pone.0276234)
Supplement: S1 File — (DOCX) [file pone.0276234.s002.docx]

**Supplementary Material**

Sex as a determinant of disease severity and clinical outcome in febrile children under five presenting to a regional referral hospital in Uganda. *McDonald, Weckman et al, 2022.*

| **Supplementary Figure 1. Flow chart of children enrolled in the study**   |
| --- |
|  |
| The flow chart presents a breakdown of participants included in the analysis by sex and age (≤1 year and >1 year). |

**Supplementary Table 1. Population parameters of disease severity at presentation by sex**

|  | | Sex Stratified | | | | | |
| --- | --- | --- | --- | --- | --- | --- | --- |
|  |  | **≤1 Year of Age (n = 758)** | | | **>1 Year of age (n = 1,291)** | | |
|  | Entire Cohort (n=2,049) | Female  (n=356) | Male  (n=402) | P Value^2^ | Female  (n=568) | Male  (n=723) | P Value^2^ |
| **Examination** |  |  |  |  |  |  |  |
| Altered Consciousness | 314 (15.3) | 61 (17.1) | 57 (14.2) | 0.255 | 92 (16.2) | 104 (14.4) | 0.383 |
| Convulsions | 378 (18.4) | 56 (15.7) | 50 (12.4) | 0.187 | 142 (25.0) | 130 (18.8) | 0.002 |
| Coma | 124 (6.1) | 28 (7.9) | 23 (5.7) | 0.247 | 32 (5.6) | 41 (5.7) | 0.950 |
| Prostration | 513 (25.0) | 103 (28.9) | 112 (27.9) | 0.758 | 134 (23.6) | 164 (22.7) | 0.695 |
| Difficulty breathing | 554 (27.0) | 123 (34.6) | 137 (34.1) | 0.869 | 131 (23.1) | 163 (22.5) | 0.830 |
| Deep breathing | 505 (24.6) | 95 (26.7) | 136 (33.8) | 0.037 | 129 (22.7) | 145 (20.1) | 0.247 |
| Nasal flaring | 459 (22.4) | 89 (25.0) | 119 (29.6) | 0.163 | 120 (21.1) | 131 (18.1) | 0.170 |
| Intercostal retractions | 460 (22.4) | 94 (26.4) | 128 (31.8) | 0.111 | 115 (20.2) | 123 (17.0) | 0.136 |
| Subcostal retractions | 412 (20.1) | 85 (23.9) | 117 (29.1) | 0.105 | 99 (17.4) | 111 (15.4) | 0.316 |
| Delayed CRT>2s | 294 (14.3) | 59 (16.6) | 56 (13.9) | 0.298 | 67 (11.8) | 112 (15.5) | 0.054 |

^1^Data are presented as median [interquartile range] or frequency (percent) as appropriate, ^2^Results of Wilcoxon rank-sum test or chi-squared test as appropriate; bolded values indicate P < 0.002 after Bonferroni correction (P = 0.05/26 tests). Abbreviations: capillary refill time (CRT).

**Supplementary Table 2. Treatment by diagnosis and sex**

|  | | Sex Stratified | | | | | |
| --- | --- | --- | --- | --- | --- | --- | --- |
|  |  | **≤1 Year of Age** | | | **>1 Year of age** | | |
| **Pneumonia** | Entire Cohort (n=329) | Female  (n=72) | Male  (n=89) | P Value^2^ | Female  (n=87) | Male  (n=81) | P Value^2^ |
| Treatment with oxygen | 70 (21.3) | 18 (25.0) | 19 (21.3) | 0.551 | 16 (18.4) | 17 (21.0) | 0.699 |
| Antibiotic  Ceftriaxone  Other | 243 (73.9)  215 (65.3) | 51 (70.8)  52 (72.2) | 68 (76.4)  62 (69.7) | 0.423  0.722 | 67 (77.0)  44 (51.7) | 57 (70.4)  57 (70.4) | 0.328  0.011 |
| **Malaria** | Entire Cohort (n=967) | Female  (n=157) | Male  (n=178) | P Value^2^ | Female  (n=272) | Male  (n=360) | P Value^2^ |
| Antimalarial  Quinine  Other | 827 (85.5)  191 (19.8) | 132 (84.1)  32 (20.4) | 165 (92.7)  26 (14.6) | 0.013  0.147 | 239 (87.9)  48 (17.6) | 291 (80.8)  85 (23.6) | 0.021  0.064 |
| Given blood transfusion | 302 (31.2) | 62 (39.5) | 64 (36.0) | 0.389 | 83 (30.5) | 93 (25.8) | 0.198 |

^1^Data are presented as frequency (percent), ^2^Results of chi-squared test; P < 0.004 would be considered significant after Bonferroni correction (P = 0.05/12)

**Supplementary Table 3. Treatment by disease severity and sex**

|  | **≤1 Year of Age** | | | | | |
| --- | --- | --- | --- | --- | --- | --- |
|  | LODS<2 (n = 614) | | | LODS≥2 (n = 144) | | |
|  | Female (n = 290) | Male (n = 324) | P Value^2^ | Female (n = 66) | Male (n = 78) | P Value^2^ |
| **Treatment**^1^ | | | | | | |
| Glucose | 54 (18.6) | 54 (16.7) | 0.498 | 30 (45.5) | 35 (44.9) | 0.878 |
| IV Fluids | 39 (13.4) | 44 (13.6) | 0.996 | 16 (24.2) | 17 (21.8) | 0.653 |
| Oxygen | 10 (3.4) | 16 (4.9) | 0.385 | 32 (48.5) | 20 (25.6) | 0.003 |
| Transfusion | 85 (29.3) | 85 (26.2) | 0.294 | 43 (65.2) | 54 (69.2) | 0.795 |
| Ceftriaxone | 139 (47.9) | 162 (50.0) | 0.730 | 47 (71.2) | 54 (69.2) | 0.687 |
| Quinine | 199 (68.6) | 230 (71.0) | 0.526 | 40 (60.6) | 50 (64.1) | 0.676 |
|  | **>1 Year of Age** | | | | | |
|  | LODS<2 (n = 1,110) | | | LODS≥2 (n = 181) | | |
|  | Female (n = 481) | Male (n = 629) | P Value^2^ | Female (n = 87) | Male (n = 94) | P Value^2^ |
| **Treatment**^1^ | | | | | | |
| Glucose | 80 (16.6) | 120 (19.1) | 0.293 | 41 (47.1) | 51 (54.3) | 0.301 |
| IV Fluids | 35 (7.3) | 58 (9.2) | 0.247 | 21 (24.1) | 20 (21.3) | 0.671 |
| Oxygen | 10 (2.1) | 19 (3.0) | 0.332 | 23 (26.4) | 26 (27.7) | 0.823 |
| Transfusion | 129 (26.8) | 165 (26.2) | 0.886 | 62 (71.3) | 63 (67.0) | 0.537 |
| Ceftriaxone | 241 (50.1) | 296 (47.1) | 0.335 | 60 (69.0) | 64 (68.1) | 0.983 |
| Quinine | 365 (75.9) | 455 (72.3) | 0.197 | 35 (40.2) | 45 (47.9) | 0.271 |

^1^Data are presented frequency (percent), ^2^Results of chi-squared test; P < 0.002 would be considered significant after Bonferroni correction (P = 0.05/24). Abbreviations: intravenous (IV); Lambaréné Organ Dysfunction Score (LODS).

**Supplementary Table 4. Analyte concentrations by disease severity and sex**

|  | **≤1 Year of Age** | | | | | |
| --- | --- | --- | --- | --- | --- | --- |
|  | LODS<2 (n = 614) | | | LODS≥2 (n = 144) | | |
|  | Female (n = 290) | Male (n = 324) | P Value^2^ | Female (n = 66) | Male (n = 78) | P Value^2^ |
| **Endothelial Activation** | | | | | | |
| sICAM-1 (ng/mL) | 630.6 [325.6, 1047.4] | 584.2 [331.4, 1008.1] | 0.654 | 970.1 [632.2, 173.3] | 990.3 [532.6, 157.0] | 0.587 |
| sVCAM-1 (ng/mL) | 3619.2 [1974.8, 6667.9] | 3574.2 [2102.2, 5611.0] | 0.788 | 5730.0 [2947.1, 8488,7] | 4221.0 [2545.6, 7761.0] | 0.297 |
| Ang-2 (ng/mL) | 8.6 [5.7, 12.3] | 7.9 [5.6, 11.4] | 0.178 | 16.1 [9.4, 22.4] | 17.2 [12.1, 21.9] | 0.475 |
| Ang-1 (ng/mL) | 2.3 [1.0, 6.3] | 2.8 [1.1, 7.2] | 0.368 | 1.6 [0.7, 3.7] | 1.2 [0.6, 3.0] | 0.413 |
| sFlt-1 (pg/mL) | 384.5 [240.2, 696.6] | 333.3 [220.2, 584.3] | 0.039 | 1156.8 [512.5, 2248.0] | 822.2 [444.0, 1485.8] | 0.074 |
| **Inflammation** | | | | | | |
| sTFNR1 (ng/mL) | 12.6 [7.3, 20.6] | 11.6 [7.5, 18.1] | 0.425 | 26.6 [10.3, 45.8] | 20.1 [12.5, 30.2] | 0.200 |
| CHI3L1 (ng/mL) | 30.1 [11.0, 61.2] | 30.7 [11.0, 54.4] | 0.832 | 81.3 [29.2, 196.6] | 80.1 [33.3, 191.1] | 0.949 |
| sTREM1 (pg/mL) | 567.4 [369.3, 790.0] | 540.3 [382.7, 798.4] | 0.657 | 1286.4 [678.0, 2434.2] | 1045.8 [703.5, 1634.9] | 0.159 |
| CXCL10 (pg/mL) | 537.1 [198.2, 1382.1] | 473.6 [164.1, 1151.3] | 0.268 | 547.6 [211.3, 1451.6] | 438.7 [179.8, 1105.5] | 0.714 |
| CXCL8, pg/mL | 15.3 [2.5, 29.9] | 12.8 [2.5, 26.3] | 0.170 | 39.8 [13.7, 190.0] | 22.7 [6.4, 147.9] | 0.054 |
| IL6 (pg/mL) | 25.1 [5, 80.7] | 22.0 [5, 77.5] | 0.309 | 129.5 [41.1, 595.5] | 57.0 [11.0, 485.3] | 0.067 |
| Granyzme B (pg/mL) | 106.5 [20, 324.2] | 113.3 [20, 294.5] | 0.575 | 243.6 [62.2, 671.8] | 131.7 [20, 356.2] | 0.133 |
|  | **>1 Year of Age** | | | | | |
|  | LODS<2 (n = 1,110) | | | LODS≥2 (n = 181) | | |
|  | Female (n = 481) | Male (n = 629) | P Value^2^ | Female (n = 87) | Male (n = 94) | P Value^2^ |
| **Endothelial Activation** | | | | | | |
| sICAM-1 (ng/mL) | 699.2 [377.9, 1169.1] | 717.4 [382.3, 1210.1] | 0.708 | 857.9 [388.3, 1317.0] | 1052.1 [492.4, 1804.6] | 0.051 |
| sVCAM-1 (ng/mL) | 4175.7 [2370.0, 6894.2] | 3938.2 [2288.1, 6458.8] | 0.272 | 4312.7 [2574.0, 9750.1] | 4603.5 [2894.2, 7276.9] | 0.887 |
| Ang-2 (ng/mL) | 7.1 [4.9, 11.3] | 6.9 [4.6, 11.1] | 0.290 | 16.1 [8.5, 29.6] | 15.6 [8.3, 23.5] | 0.269 |
| Ang-1 (ng/mL) | 2.4 [1.0, 5.3] | 3.1 [1.3, 6.8] | 0.004 | 1.8 [0.8, 3.4] | 1.9 [0.8, 4.1] | 0.652 |
| sFlt-1 (pg/mL) | 320.8 [207.1, 689.6] | 312.2 [206.0, 589.8] | 0.301 | 1101.2 [508.1, 2169.8] | 1072.4 [408.0, 2368.5] | 0.904 |
| **Inflammation** | | | | | | |
| sTFNR1 (ng/mL) | 10.6 [7.3, 17.2] | 11.4 [7.4, 16.6] | 0.453 | 17.4 [10.6, 30.2] | 18.1 [10.9, 32.7] | 0.655 |
| CHI3L1 (ng/mL) | 41.7 [11.0, 91.2] | 48.0 [21.3, 12.1] | 0.013 | 74.9 [42.0, 346.3] | 94.1 [33.8, 376.7] | 0.568 |
| sTREM1 (pg/mL) | 523.5 [355.9, 780.2] | 495.5 [338.1, 736.6] | 0.176 | 1003.6 [755.5, 2034.5] | 965.2 [604.2, 2010.6] | 0.554 |
| CXCL10 (pg/mL) | 423.2 [190.1, 1109.8] | 405.2 [150.1, 1024.1] | 0.203 | 422.2 [137.5, 1038.1] | 292.3 [142.2, 915.0] | 0.590 |
| CXCL8 (pg/mL) | 11.5 [2.5, 27.2] | 12.1 [2.5, 29.5] | 0.149 | 46.8 [8.2, 186.4] | 52.6 [9.9, 116.0] | 0.875 |
| IL6 (pg/mL) | 29.5 [5, 116.1] | 26.8 [5, 94.4] | 0.430 | 93.7 [25.3, 497.4] | 110.8 [37.2, 544.3] | 0.657 |
| Granyzme B (pg/mL) | 76.8 [20, 232.9] | 66.7 [20, 199.1] | 0.271 | 75.1 [20, 204.3] | 60.0 [20, 221.7] | 0.763 |

^1^Data are presented frequency (percent), ^2^Results of chi-squared test; P < 0.001 would be considered significant after Bonferroni correction (P = 0.05/48). Abbreviations: angiopoietin-1 (Angpt-1); angiopoietin-2 (Angpt-2); chitinase-3-like-1 protein (CHI3L1); interferon-gamma-inducible protein-10/C-X-C motif chemokine ligand 10 (IP-10/CXCL10); interleukin 6 (IL-6); interleukin-8/C-X-C motif chemokine ligand 8 (IL-8/CXCL8); Lambaréné Organ Dysfunction Score (LODS); soluble fms-like tyrosine kinase-1 (sFlt-1); soluble intracellular adhesions molecule-1 (sICAM-1); soluble triggering receptor expressed on myeloid cells-1 (sTREM-1), soluble tumor necrosis factor receptor-1 (sTNFR-1); vascular cell adhesion molecule (sVCAM-1).

**Supplementary Table 5. Analyte concentrations by in-hospital mortality and sex**

|  | **≤1 Year of Age** | | | | | |
| --- | --- | --- | --- | --- | --- | --- |
|  | Survived (n = 717) | | | Death In-Hospital (n = 41) | | |
|  | Female (n = 334) | Male (n = 383) | P Value^2^ | Female (n = 22) | Male (n = 19) | P Value^2^ |
| **Endothelial Activation** | | | | | | |
| sICAM-1 (ng/mL) | 660.4 [343.4, 1099.6] | 648.9 [362.1, 1112.1] | 0.874 | 1014.1 [506.1, 2290.0] | 799.1 [283.6, 1341.2] | 0.245 |
| sVCAM-1 (ng/mL) | 3746.8 [2055.3, 6761.7] | 3678.7 [2121.0, 6015.2] | 0.643 | 6517.1 [2626.5, 12568.8] | 4003.6 [2917.7, 9589.5] | 0.583 |
| Ang-2 (ng/mL) | 8.9 [5.9, 13.5] | 8.7 [5.9, 13.5] | 0.750 | 20.4 [15.1, 36.6] | 20.3 [13.9, 36.0] | 0.657 |
| Ang-1 (ng/mL) | 2.3 [0.9, 5.9] | 2.3 [0.9, 6.5] | 0.832 | 0.86 [0.6, 1.9] | 1.2 [0.5, 2.5] | 0.794 |
| sFlt-1 (pg/mL) | 403.3 [249.9, 816.5] | 370.7 [229.7, 702.4] | 0.102 | 1527.3 [970.7, 3358.7] | 827.3 [348.1, 2857.6] | 0.143 |
| **Inflammation** | | | | | | |
| sTFNR1 (ng/mL) | 13.2 [7.5, 23.3] | 12.6 [7.9, 20.3] | 0.472 | 29.5 [11.4, 52.0] | 20.5 [10.3, 58.5] | 0.657 |
| CHI3L1 (ng/mL) | 32.8 [11.0, 72.8] | 32.4 [11.0, 66.9] | 0.934 | 137.0 [39.2, 455.2] | 172.3 [54.2, 379.6] | 0.865 |
| sTREM1 (pg/mL) | 603.6 [394.7, 913.3] | 595.6 [393.1, 849.7] | 0.808 | 1692.5 [1073.5, 3640.1] | 1871.8 [1024.4, 2794.0] | 0.657 |
| CXCL10 (pg/mL) | 546.7 [198.9, 1382.1] | 477.5 [165.1, 1197.9] | 0.258 | 376.7 [113.9, 1945.2] | 370.2 [203.1, 700.3] | 0.906 |
| CXCL8, pg/mL | 15.8 [7.2, 33.9] | 13.1 [2.5, 29.7] | 0.067 | 145.0 [30.3, 1377.6] | 44.2 [11.8, 522.2] | 0.272 |
| IL6 (pg/mL) | 29.4 [5.0, 97.5] | 24.2 [5.0, 93.0] | 0.173 | 304.2 [81.5, 2894.6] | 57.7 [5.0, 2398.7] | 0.437 |
| Granyzme B (pg/mL) | 119.1 [20.0, 358.3] | 119.1 [20.0, 309.5] | 0.374 | 92.4 [20.0, 564.6] | 20.0 [20.0, 269.0] | 0.122 |
|  | **>1 Year of Age** | | | | | |
|  | Survived (n = 1235) | | | Death In-Hospital (n = 56) | | |
|  | Female (n = 548) | Male (n = 687) | P Value^2^ | Female (n = 20) | Male (n = 36) | P Value^2^ |
| **Endothelial Activation** | | | | | | |
| sICAM-1 (ng/mL) | 721.6 [382.0, 11196.9] | 728.1 [386.7, 1235.3] | 0.671 | 622.3 [341.6, 1435.6] | 1541.6 [662.8, 3195.3] | 0.035 |
| sVCAM-1 (ng/mL) | 4171.7 [2370.9, 7041.9] | 3967.7 [2331.4, 6506.8] | 0.259 | 5208.8 [2884.5, 12928.2] | 5174.4 [2684.6, 11645.3] | 0.745 |
| Ang-2 (ng/mL) | 7.6 [5.1, 13.1] | 7.2 [4.6, 12.0] | 0.056 | 18.2 [11.8, 26.3] | 18.4 [12.6, 28.7] | 0.946 |
| Ang-1 (ng/mL) | 2.3 [1.0, 4.9] | 2.9 [1.3, 6.3] | 0.0013 | 1.2 [0.8, 3.0] | 1.5 [0.8, 2.7] | 0.966 |
| sFlt-1 (pg/mL) | 370.0 [216.5, 763.6] | 322.3 [212.5, 640.3] | 0.080 | 1557.8 [804.7, 2980.3] | 1524.0 [675.0, 3762.7] | 0.932 |
| **Inflammation** | | | | | | |
| sTFNR1 (ng/mL) | 11.1 [7.4, 17.6] | 11.6 [7.6, 17.1] | 0.679 | 36.2 [23.4, 51.5] | 31.9 [17.6, 46.6] | 0.442 |
| CHI3L1 (ng/mL) | 43.3 [11.0, 97.0] | 48.9 [21.3, 129.2] | 0.035 | 331.4 [73.2, 539.1] | 428.5 [67.7, 1026.8] | 0.791 |
| sTREM1 (pg/mL) | 565.8 [367.6, 845.6]] | 511.8 [341.8, 763.9] | 0.018 | 2034.4 [1146.9, 2850.5] | 1953.2 [905.2, 2823.9] | 0.784 |
| CXCL10 (pg/mL) | 419.1 [178.1, 1109.8] | 401.0 [149.1, 1007.6] | 0.244 | 499.0 [225.8, 1332.1] | 261.6 [142.9, 1084.7] | 0.274 |
| CXCL8 (pg/mL) | 11.6 [2.5, 31.8] | 12.3 [2.5, 32.2] | 0.240 | 327.6 [61.1, 2465.9] | 114.8 [43.0, 671.1] | 0.356 |
| IL6 (pg/mL) | 34.6 [5.0, 122.8] | 28.9 [5.0, 101.4] | 0.335 | 1556.7 [158.0, 11137.5] | 522.3 [99.0, 5635.2] | 0.308 |
| Granyzme B (pg/mL) | 76.4 [20.0, 214.6] | 66.7 [20.0, 203.7] | 0.470 | 159.4 [20.0, 585.5] | 20.0 [20.0, 405.8] | 0.421 |

^1^Data are presented frequency (percent), ^2^Results of chi-squared test; P < 0.001 would be considered significant after Bonferroni correction (P = 0.05/48). Abbreviations: angiopoietin-1 (Angpt-1); angiopoietin-2 (Angpt-2); chitinase-3-like-1 protein (CHI3L1); interferon-gamma-inducible protein-10/C-X-C motif chemokine ligand 10 (IP-10/CXCL10); interleukin 6 (IL-6); interleukin-8/C-X-C motif chemokine ligand 8 (IL-8/CXCL8); soluble fms-like tyrosine kinase-1 (sFlt-1); soluble intracellular adhesions molecule-1 (sICAM-1); soluble triggering receptor expressed on myeloid cells-1 (sTREM-1), soluble tumor necrosis factor receptor-1 (sTNFR-1); vascular cell adhesion molecule (sVCAM-1).
